# Construction and Validation of an Autophagy-Related Prognostic Signature and a Nomogram for Bladder Cancer

**DOI:** 10.3389/fonc.2021.632387

**Published:** 2021-06-18

**Authors:** Xin Yan, Hua-Hui Wu, Zhao Chen, Guo-Wei Du, Xiao-Jie Bai, Kurerban Tuoheti, Tong-Zu Liu

**Affiliations:** Department of Urology, Zhongnan Hospital of Wuhan University, Wuhan, China

**Keywords:** autophagy-related prognostic signature, bladder cancer, bioinformatics, nomogram, prognosis, WGCNA

## Abstract

**Objective:**

Bladder cancer (BC) is one of the top ten cancers endangering human health but we still lack accurate tools for BC patients’ risk stratification. This study aimed to develop an autophagy-related signature that could predict the prognosis of BC. In order to provide clinical doctors with a visual tool that could precisely predict the survival probability of BC patients, we also attempted to establish a nomogram based on the risk signature.

**Methods:**

We screened out autophagy-related genes (ARGs) combining weighted gene co-expression network analysis (WGCNA) and differentially expressed gene (DEG) in BC. Based on the screened ARGs, we performed survival analysis and Cox regression analysis to identify potential prognostic biomarkers. A risk signature based on the prognostic ARGs by multivariate Cox regression analysis was established, which was validated by using seven datasets. To provide clinical doctors with a useful tool for survival possibility prediction, a nomogram assessed by the ARG-based signature and clinicopathological features was constructed, verified using four independent datasets.

**Results:**

Three prognostic biomarkers including BOC (*P* = 0.008, HR = 1.104), FGF7(*P* = 0.030, HR = 1.066), and MAP1A (*P* = 0.001, HR = 1.173) were identified and validated. An autophagy-related risk signature was established and validated. This signature could act as an independent prognostic feature in patients with BC (*P* = 0.047, HR = 1.419). We then constructed two nomograms with and without ARG-based signature and subsequent analysis indicated that the nomogram with ARG signature showed high accuracy for overall survival probability prediction of patients with BC (C-index = 0.732, AUC = 0.816). These results proved that the ARG signature improved the clinical net benefit of the standard model based on clinicopathological features (age, pathologic stage).

**Conclusions:**

Three ARGs were identified as prognosis biomarkers in BC. An ARG-based signature was established for the first time, showing strong potential for prognosis prediction in BC. This signature was proven to improve the clinical net benefit of the standard model. A nomogram was established using this signature, which could lead to more effective prognosis prediction for BC patients.

## Introduction

As the most common malignancy of the urinary system, bladder cancer (BC) has six pathological types, with bladder urothelial carcinoma the most common pathological type (1, 2). According to research, there were 549,393 new cases worldwide in 2018 (3, 4). As the American Cancer Society has reported, there will be approximately 81,400 new BC cases diagnosed, and 17,980 new BC deaths in the USA in 2020 (3). Patients with BC also occupy poor prognosis, and the survival rate of five years has not raised significantly with the improvement of early diagnosis and therapy of BC (2). Even now the prognosis of tumor patients still depends on TNM staging (5). However, some limitations exist in TNM staging (6) as it sometimes might be not accurate in the prediction of the prognosis of patients, such as the same TNM staging may also have completely different prognostic results (6). Thus, the discovery of novel methods for predicting the prognosis of BC patients more precisely is the first requirement.

Autophagy is an active method of cell death, whose role is to maintain the balance of synthesis and degradation of components in cells (7). Some studies have concluded that there is a close association between autophagy and tumors (8). More concretely, autophagy could clear or inhibit newly formed tumor cells (9). It could also promote the development and progression of tumors after tumor formation (9, 10).

Recently, some studies have demonstrated that modulation of autophagy could improve the sensitivity of BC tumors to chemotherapeutic agents (11). More and more studies have focused on exploring the association between autophagy and the prognosis of BC (12). However, to the best of our knowledge, none of them are attempting to establish a risk signature for prognosis of BC patient prediction, by using autophagy-related genes (ARGs) (13, 14). The aim of the present study was to construct an autophagy-related signature that could accurately act as a prognosis prediction tool in BC.

This study first screened out 37 ARGs among 490 ARGs by using weighted gene co-expression network analysis (WGCNA) (15) and differentially expressed gene (DEG) identification. Nine among the 37 ARGs were significantly related to the survival of BC patients by preforming overall survival (OS) and disease-free survival (DFS) analyses. Three from the nine ARGs showed a strong correlation with the prognosis of the BC patients who were then screened. We developed a risk signature based on the three ARGs, which was positively associated with bladder cancer invasiveness and might significantly forecast the prognosis of BC. Finally, based on these clinical features, two nomograms with and without the risk signature were established separately. The nomogram with the risk signature established for the survival rate of BC patient prediction could provide guidance for clinical practice.

## Materials and Methods

### BC Studies Collection

A flow diagram of our research process was shown in **Figure S1**. Bladder cancer microarray data (TCGA-BLCA data) displayed as count number was first retrieved from The Cancer Genome Atlas (TCGA) database (https://genomecancer.ucsc.edu/). Samples without complete clinical information were regarded as substandard samples in the present study. After excluding substandard samples, 427 samples including 408 BC samples and 19 normal tissues were included in the following research. We also retrieved the related clinical information including age, gender, histologic grade, pathologic stage, follow-up time, and survival state.

The TCGA-BLCA data was firstly preprocessed by using the R package “DEseq.2” before using the data (16). The methods included normalization and log2 transformation. Seven datasets were retrieved from public databases for validation. Among them, four independent GEO datasets [GSE13507 (17, 18), GSE19915 (19), GSE31684 (20, 21), GSE32894 (22)] were retrieved from the Gene Expression Omnibus (GEO) database (http://www.ncbi.nlm.nih.gov/geo/). GSE13507, the platform of which was GPL6102, included 10 normal bladder mucosae and 165 bladder cancer tissues. GSE19915, performed on GPL3883, contained 142 BCs. GSE31684 including 93 bladder cancer tissues was obtained from the GPL570 platform. GSE32894, the platform of which was GPL6947, contained 308 BC tissues. The related survival information was also downloaded in this study. For the four datasets shown as raw expression data, normalization and transformation were performed by using the R package “affy” (23). In addition, the expression data of the IMvogor210 (24) cohort displayed as a count number was immediately retrieved from http://research-pub.Gene.com/imvigor210corebiologies. We then transformed the count value into the TPM value by using the R package “DEseq2”. After that, a total of 298 samples were included for subsequent analysis. Another independent cohort E-MTAB-4321 (25) containing 476 BC samples was also collected from the ArrayExpress database (https://www.ebi.ac.uk/arrayexpress/). The normalization expression matrix of E-MTAB-4321 was retrieved from this database directly. Moreover, because of the strong association between molecular subtypes of BLCA and tumor invasiveness, we retrieved another dataset E-MTAB-1803 (26, 27). We directly downloaded the normalization expression matrix of this dataset. In total, 85 bladder cancer samples with complete molecular subtype information were included in the present study.

### Autophagy-Related Gene Collection

Based on previous studies, ARGs were collected based on Human Autophagy Database (28) (HADb, http://www.autophagy.lu/index.html) and the GO_AUTOPHAGY gene set. The GO_AUTOPHAGY gene set was downloaded from GSEA (29) website (http://software.broadinstitute.org/gsea/index.jsp). A total of 531 ARGs were obtained from the two databases, 490 of which with available expression values in TCGA-BLCA data were selected for the present study.

### Weighted Co-Expression Network Construction

Weighted co-expression network was constructed by using the R package “WGCNA” (30) based on the 490 ARGs collected from previous analyses. Firstly, gsg (goodSamplesGenes) and sample network methods were used to check the expression data profile of the 490 ARGs from TCGA-BLCA data, to validate whether they were good samples or good genes. Z.Ku was calculated by the following formula: Z.ku = (ku-mean(k))/(sqrt(var(k))). In this study, samples with Z.Ku < -2.5 were excluded from WGCNA. β (soft threshold power beta) was then chosen under the control of scale free topology criterion. Furthermore, adjacency was transformed into TOM, and genes were assorted into gene modules based on obtaining branch cutting methods using the following indexes: minClusterSize = 30, and deepSplit = 2. To merge modules with high correlation, a cut line was also set (correlation ≥ 0.75) by reckoning the dissimilarity of module eigengenes (MEs).

### Disease-Related Module Identification

After identifying modules formed by genes, we calculated the Module Significance (MS) to quantify the module eigengene in relationship with trait. In this study, we focused on the disease status (BC or normal). Thus, in the present study, the most positive correlation module and the most negative correlation module were identified as disease-related modules. All the genes in the disease-related modules were included for subsequent analysis.

### Differentially Expressed Gene (DEG) Identifying and Enrichment Analyses

In this study, the DEGs between normal tissues and BC tissues were identified by using the R package “edgeR” (31). We set the standards of adjusted *P* value < 0.05 and |log2FC| ≥ 1.0 for the identification of differentially expressed autophagy-related gene (ARG). Furthermore, we overlapped genes between genes in the disease-related modules and DEGs. To better understand the capacity functions of these genes, Gene Ontology (GO) enrichment analysis and Kyoto Encyclopedia of Genes and Genomes (KEGG) pathway enrichment analysis were performed by R package “clusterProfiler” (32). *P* < 0.05 was set as the cut-off criterion for both the GO and KEGG analyses. R package “GOplot” (33) was used for visualization.

### Potential Prognostic Gene Identification and mRNA Expression Level Validation

Genes overlapped between genes in the disease-related modules and differentially expressed genes (DEGs) were validated to see if they were potential prognostic biomarkers based on Gene Expression Profiling Interactive Analysis (GEPIA) (34) (http://gepia.cancer-pku.cn/). According to the gene expression levels, the samples were divided into two groups (high- expression group and low- expression group) in TCGA-BLCA data by using GEPIA (the median expression of each gene was selected as grouping cut-off criterion). Two survival analysis types (overall survival (OS) and disease-free survival (DFS)) were performed and genes showed significant *p* values (*p* < 0.05) in both the two analysis types were regarded as potential prognostic biomarkers in this study. Furthermore, by using GSE13507 and TCGA-BLCA data, we compared the mRNA expression of these potential prognostic ARGs in BC samples and normal tissues.

### Establishment of an ARG-Based Risk Signature

In this study, we obtained prognostic ARGs among potential prognostic genes by conducting a univariate Cox analysis of OS. ARGs with *P* < 0.05 were immediately used for multivariate Cox analysis. We regarded ARGs with *P* < 0.05 in multivariate Cox analysis as prognostic genes. Furthermore, based on the regression coefficient (Coef) and gene expression values, an autophagy-related prognostic signature was constructed. The risk score (RS) of each BC sample was calculated based on the following formula:

$$\text{Risk}\;\text{score}=\Sigma_{i=1}^{n}Coef_{i}\times Exp_{i}$$

In which Coef is the regression coefficient and Exp represents the expression value of each prognostic ARG. To validate the prognostic value of the autophagy-related prognostic signature, the risk score of each BC sample in TCGA-BLCA data, GSE13507, GSE19915, GSE31684, GSE32894, E-MTAB-4321, and IMvigor210 was calculated based on this formula. In each dataset, the samples were divided into high- and low-risk groups by setting the centermost element of the RS as the standard for grouping. By using the R package “survival” (35), immediately, survival analysis of the two groups was conducted (GSE13507: OS, cancer-specific survival (CSS); GSE19915: CSS, progression-free survival (PFS); GSE31684: OS, CSS; GSE32894: CSS, PFS; E-MTAB-4321: PFS; IMvigor210: OS; TCGA-BLCA: OS, DFS). In addition, the time-dependent (1-, 3-, 5-year) receiver operating characteristic (ROC) analysis was performed based on “survivalROC” (36) in R software.

### Cox Proportional Hazards Regression Analysis

To verify the prognostic value of the prognostic signature, we included the risk score of this ARG-based signature and some important clinicopathological factors (gender, age, pathologic stage, and histologic grade) for univariable Cox analysis of OS based on TCGA-BLCA data. To check whether this gene signature was irrelevant to other clinicopathological factors for OS prediction of BC, we included the factors (*P* < 0.05) for multivariate Cox analysis. Moreover, by using TCGA-BLCA data, we also performed univariable and multivariate Cox analyses of DFS *via* the same method. Visualization was finished by using the R package “forestplot” (37).

### Nomogram Construction and Validation

After performing cross-validation (which could avoid the over-fitting problem), we immediately used the R package “rms” to establish nomograms with or without the ARG-based signature. To test the nomograms, we also plotted the calibrate curve, the 45° line that represents the best prediction. The consistency index (C-index) between actual probability and predicted probability was measured to evaluate the prediction effectiveness of the nomograms and t. ROC curves were also plotted using the R package “pROC” (38). In addition, we performed time-dependent (1-, 3-, 5-year) receiver operating characteristic (ROC) analysis to check the stability of the nomogram with and without ARG signature. Moreover, we used R package “rmda” (39) to perform decision curve analysis (DCA) and examine the value of the signature in clinical applications. We evaluated the clinical net benefit using the nomogram with and without ARG signature for predicting 1-, 3-, 5- year survival probability. In the present study, TCGA-BLCA data, GSE31684, GSE13507, and IMvigor210 with complete OS information were included for internal verification and external verification.

### Gene Set Enrichment Analysis (GSEA)

To better understand the lurking functions of the autophagy-related prognostic signature, we evaluated the median RS by using TCGA-BLCA data. After that, 408 BCs were split into two groups accurately (high-risk group: n = 204; low-risk group: n = 204). “c2.cp.kegg.v7.3.symbols.gmt” was set as the reference gene sets. GSEA (29) was conducted between the two groups. In this study, KEGG signaling pathways reached the standards (nominal *P* < 0.05, |ES| > 0.6, gene size ≥ 100 and FDR < 25%) were significantly enriched.

## Results

### Identification of Two Disease-Related Modules in a Weighted Co-Expression Network

**Figure 1:**
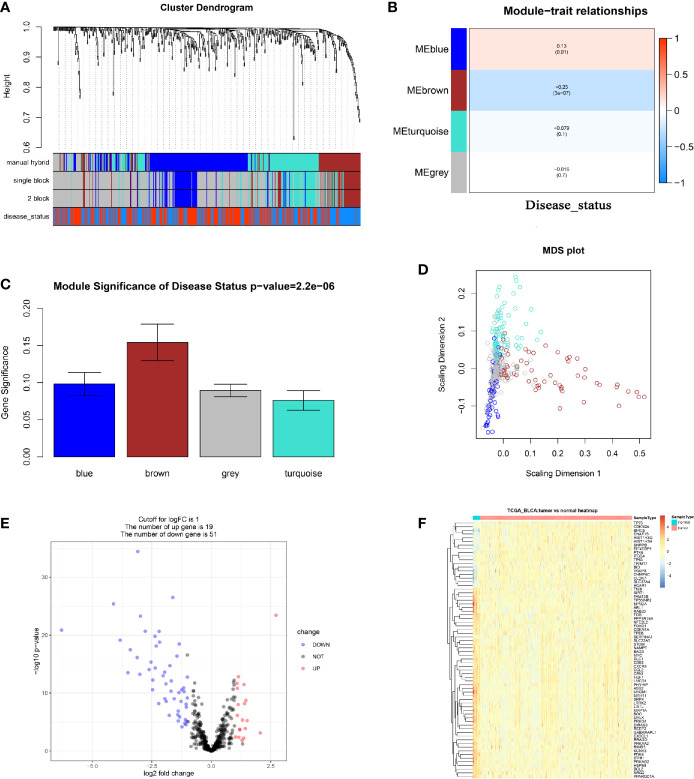
Relevant module associated with clinical information identification and differentially expressed ARGs identification. **(A)** Dendrogram of all differentially expressed genes clustered based on a dissimilarity measure (1-TOM). Manual hybrid: the manual (interactive) branch cutting approach; single block: the automatic single block analysis; 2 block: the 2 block analysis; disease status: the trait interested us most (BLCAs or Normal tissues). **(B)** Heatmap of the correlation between module eigengenes and different clinical information of BLCA (disease status). **(C)** Distribution of average gene significance and errors in the modules associated with disease status of BLCA. **(D)** Classical MDS plot whose input is the TOM dissimilarity. Each dot (gene) is colored by the module assignment. **(E)** Volcano plot visualizing differentially expressed ARGs in TCGA-BLCA data. **(F)** Heatmap of differentially expressed ARGs between tumor samples *vs* normal samples (P < 0.05, fold change > 1, TCGA-BLCA).

In total, 18 outlier samples of the 428 samples from the TCGA database were identified, which were excluded for subsequent analysis (**Figure S2**). They were then combined with ARGs, and we constructed a weighted co-expression network by using the R package “WGCNA”. Beta (β) = 4 (scale free R2 = 0.85) was chosen as the soft-thresholding for adjacencies calculation (**Figure S3**). The 490 ARGs were assigned to three modules (**Figure 1A**). In the present study, brown module (*P* = 3E-07, *r* = -0.25) showed the most negative correlation with disease status, meanwhile the blue module (*P* = 0.01, *r* = 0.13) showed the most positive correlation with disease status (**Figure 1B**). Moreover, the MS of the two modules was the highest of any other modules (**Figure 1C**). Thus, we regarded the brown module and blue module as disease-related modules in this study. In total, 123 ARGs including 62 ARGs in the blue module and 61 ARGs in the brown module were included for subsequent analysis. We also plotted a multidimensional scaling (MDS) plot for bio-similarity of modules estimation, which demonstrated that the three modules were distinguished well (**Figure 1D**).

### Differentially Expressed ARGs in BC

We screened out 70 differentially expressed ARGs including 19 up-regulated ARGs and 51 down-regulated ARGs *via* R package “edgeR” (**Figure 1E**). We also showed a heatmap of DEGs as a part of the result (**Figure 1F**). The detailed information of each differentially expressed ARG was shown in **Table S1**. Furthermore, 37 ARGs overlapping between ARGs in the disease-related modules and differentially expressed ARGs were obtained (**Figure S4A**). To understand the lurking function of the 37 ARGs, GO and KEGG enrichment analyses were carried out. These ARGs were sufficiently enriched in 53 BPs (**Table S2**), the top 10 of which were autophagy, response to starvation, cellular response to drug, macroautophagy, intrinsic apoptotic signaling pathway in response to endoplasmic reticulum stress, regulation of autophagy, response to hypoxia, a process utilizing autophagic mechanism, positive regulation of autophagy, and response to decreased oxygen levels (**Figure S4B**). As for the KEGG pathway analysis, the 37 ARGs were significantly enriched in longevity regulating pathway, glucagon signaling pathway, AMPK signaling pathway, platelet activation, vascular smooth muscle contraction, autophagy – animal, and apelin signaling pathway (**Figure S4C**).

### Nine ARGs Were Screened Out as Potential Prognostic Biomarkers

**Figure 2:**
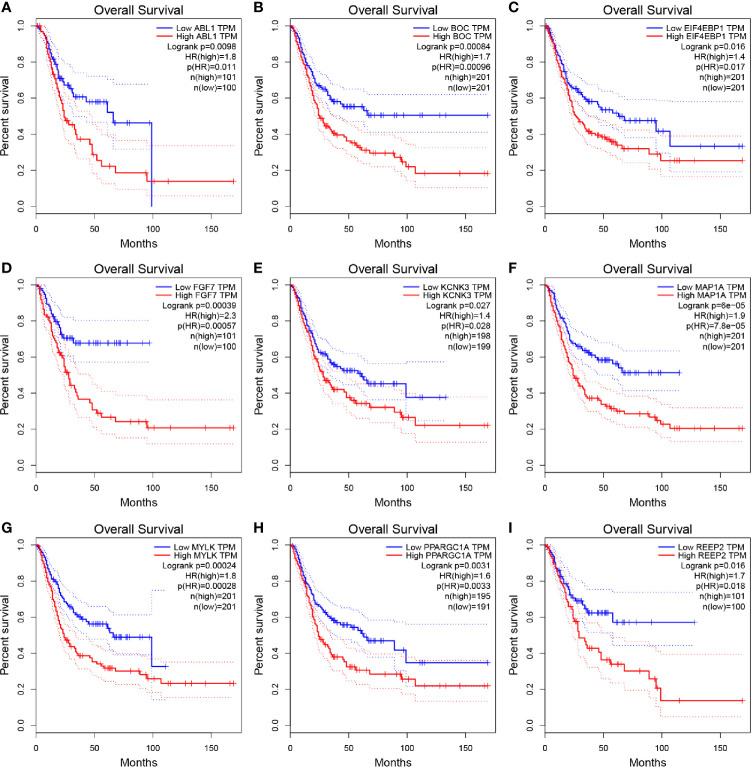
Kaplan–Meier overall survival (OS) curves for BLCA patients assigned to groups of high and low expression level based on the nine genes, respectively. (**A–I** show the results of ABL1, BOC, EIF4EBP1, FGF7, KCNK3, MAP1A, MYLK, PPARGC1A, REEP2, respectively).

Based on the 37 ARGs identified before, OS and DFS analyses were performed to carry out the correlation between ARGs and survival (**Table S3**). In total, nine genes including ABL1 (ABL proto-oncogene 1, non-receptor tyrosine kinase), BOC (BOC cell adhesion associated, oncogene regulated), EIF4EBP1 (eukaryotic translation initiation factor 4E binding protein 1), FGF7 (fibroblast growth factor 7), KCNK3 (potassium two pore domain channel subfamily K member 3), MAP1A (microtubule associated protein 1A), MYLK (myosin light chain kinase), PPARGC1A (PPARG coactivator 1 alpha), and REEP2 (receptor accessory protein 2) were determined to be associated with OS (**Figure 2**) and DFS (**Figure S5**) of BC patients. Then, as shown in **Figure S6A**, ABL1, BOC, FGF7, KCNK3, MAP1A, MYLK, PPARGC1A, and REEP2 were validated to be significantly higher expressed in normal tissues compared with BLCA tissues. By contrast, EIF4EBP1 was significantly higher expressed in BLCA tissues compared to normal tissues. A similar result was concluded by using dataset GSE13507 (**Figure S6B**).

### An Autophagy-Related Risk Signature Was Developed *via* Potential Prognostic Biomarkers

**Figure 3:**
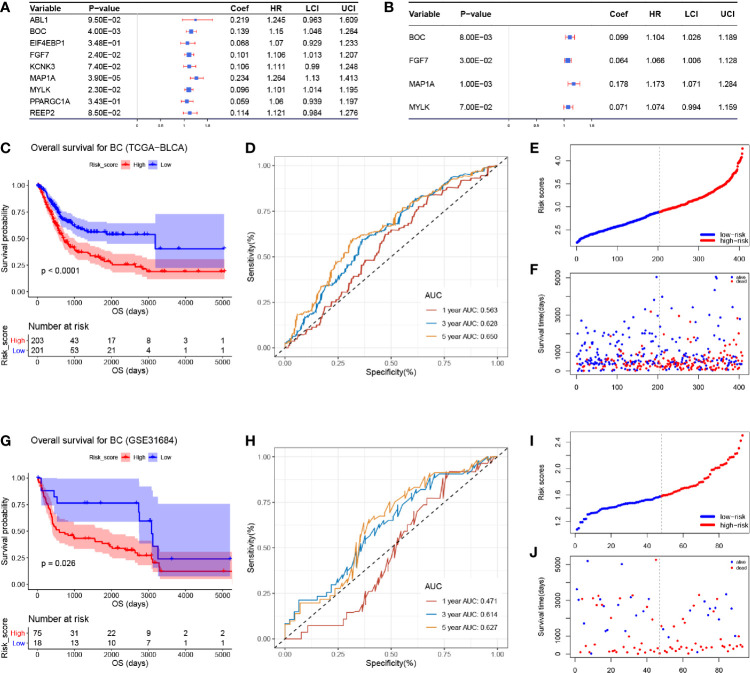
Cox proportional hazards regression analysis and the correlation between the three-gene autophagy-related signature for OS and the prognosis of patients with BC. **(A)** Univariate Cox regression analysis of the nine differentially expressed autophagy-related genes. **(B)** Multivariate Cox regression analysis of the four autophagy-related genes. **(C)** Kaplan-Meier OS curves for the high- and low-risk groups by using TCGA-BLCA data. **(D)** ROC curve indicating the predictive accuracy of the autophagy-related signature for OS by using TCGA-BLCA data. **(E)** Distribution of the risk scores of BC patients based on TCGA-BLCA data. **(F)** The number of survivors and non-survivors with different risk scores based on TCGA-BLCA data; red represents the number of non-survivors, and blue represents the number of survivors. **(G)** Kaplan-Meier OS curves for the high- and low-risk groups by using GSE31684 data. **(H)** ROC curve indicating the predictive accuracy of the autophagy-related signature for OS by using GSE31684. **(I)** Distribution of the risk scores of BC patients based on GSE31684. **(J)** The number of survivors and non-survivors with different risk scores based on GSE31684; red represents the number of non-survivors, and blue represents the number of survivors.

We immediately performed univariate Cox analysis of the OS for the nine ARGs (**Figure 3A**). Four genes including BOC, FGF7, MAP1A, and MYLK were then used for multivariate Cox analysis. Three genes including BOC (*P* = 0.008, Coef = 0.009, HR = 1.104), FGF7 (*P* = 0.030, Coef = 0.064, HR = 1.066), and MAP1A (*P* = 0.001, Coef = 0.178, HR = 1.173) were immediately identified for risk signature construction (**Figure 3B**). We calculated the risk score as follows:

$$\text{Risk}\;\text{score}=0.099\times \text{Ex}{\text{p}}_{BOC}+0.064\times \text{Ex}{\text{p}}_{FGF7}+0.178\times \text{Ex}{\text{p}}_{MAP1A}.$$

The risk score of each sample in TCGA-BLCA data is shown in **Table S4**. We divided 408 BC samples into a high-risk group (n = 204) and a low-risk group (n = 204) according to the median value of risk score in TCGA-BLCA data. Further analysis demonstrated that BC patients in the high-risk group occupied a worse OS (**Figure 3C**, *P* < 0.0001). Moreover, by using TCGA-BLCA data, the prognostic accuracy of the risk score model was 0.563 at 1 year, 0.628 at 3 years, and 0.650 at 5 years, accurately (**Figure 3D**). We visualized the risk score of BC patients in TCGA-BLCA data (**Figure 3E**). The number of patients who died in the high-risk group increased compared with the low-risk group (**Figure 3F**).

### Validation of the Three-Gene Based Prognostic Signature

To validate the robustness of the risk signature, the RS for each BC patient in GSE31684 was also evaluated (**Table S5**). BC tissues were split into the high- (n = 46) and low-risk group (n = 47) as previously described. Based on GSE31684, we reached the same conclusion, that BC patients in the high-risk group had worse OS compared with patients in the low-risk group, respectively (**Figure 3G**, *P* = 0.026). The AUC values of 1-year, 3-years, and 5-years OS were 0.471, 0.614, and 0.627 by using GSE31684, separately (**Figure 3H**). The risk scores of each BC patient in GSE31684 are visualized as **Figure 3I**, similarly, an increasing number of BC patients died as the risk score increased (**Figure 3J**).

The RS for each patient in IMvigor210 (**Table S6**) and GSE13507 (**Table S7**) were also calculated, with similar results to those described previously. BC patients with a higher risk score had a worse OS compared to those with a low risk score (IMvigor210: *P* = 0.013, **Figure S7A**; GSE13507: *P* = 0.072, **Figure S7C**). The AUC values of 1-year, 3-years, 5-years OS were 0.587, 0.647, and 0.670 were calculated using IMvigor210 (**Figure S7B**) and 0.604, 0.583, and 0.563 by using GSE13507 (**Figure S7D**) separately. We also validated this risk signature when setting CSS, PFS, or DFS as an endpoint.

The risk scores of each BC sample in GSE19915 (**Table S8**), GSE32894 (**Table S9**), and E-MTAB-4321 (**Table S10**) were also explored with the same formula. The BC patient with worse CSS, significantly, were from the high-risk group (GSE13507: *P* = 0.011, **Figure S7E**; GSE19915: *P* = 0.030, **Figure S7G**; GSE31684: *P* = 0.017, **Figure S7I**; GSE32894: *P* < 0.001, **Figure S7K**). Furthermore, the AUCs for the 1-year, 3-years, and 5-years CSS of GSE13507 was 0.678, 0.679, and 0.679, accurately (**Figure S7F**). The AUCs for the 1-year, 3-years, and 5-years CSS of GSE19915 were 0.555, 0.748, and 0.748 (**Figure S7H**). The AUCs for the 1-year, 3-years, and 5-years CSS of GSE31684 were 0.518, 0.657, and 0.668, accurately (**Figure S7J**). The AUCs for the 1-year, 3-years, and 5-years CSS of GSE32894 were 0.638, 0.711, and 0.765, accurately (**Figure S7L**).

By setting PFS as the endpoint, we also found that BC patients in the low-risk group were determined to have better PFS compared with patients in the high-risk group (GSE19915: *P* = 0.033, **Figure S7M**; GSE32894: *P* = 0.003, **Figure S7O**; E-MTAB-4321: *P* = 0.007, **Figure S7Q**). The AUCs for the 1-year, 3-years, and 5-years PFS of GSE19915 were 0.571, 0.722, and 0.759, accurately (**Figure S7N**). The AUCs for the 3-years and 5-years PFS of GSE32894 was 0.679, and 0.898, accurately (**Figure S7P**). The AUCs for the 1-year, 3-years, and 5-years PFS of E-MTAB-4321 were 0.515, 0.621, and 0.582, accurately (**Figure S7R**). As shown in **Figure S9**, BC patients in the low-risk group also had better DFS compared with those in the high-risk group (*P* < 0.001, **Figure S7S**). The AUCs for the 1-year, 3-years, and 5-years DFS of TCGA-BLCA data were 0.627, 0.623, and 0.578, accurately (**Figure S7T**).

### Validating the Autophagy-Related Signature as an Independent Prognostic Feature for BC Patients

**Figure 4:**
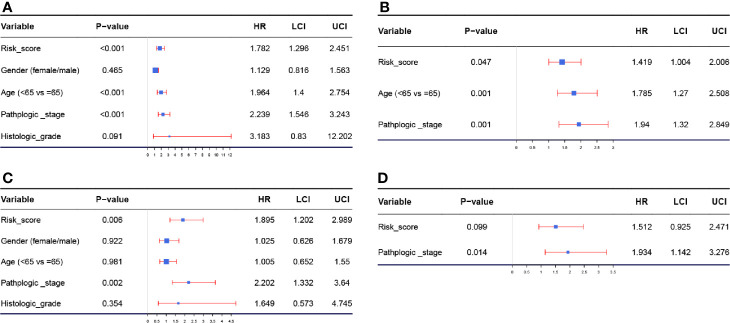
**(A)** Forest plot summary of analyses of OS univariate analysis of Risk score, gender, age, pathologic stage, and histologic grade by using TCGA-BLCA data. **(B)** Forest plot summary of analyses of OS multivariate analysis of Risk score, age, and pathologic stage by using TCGA-BLCA data. **(C)** Forest plot summary of analyses of DFS univariate analysis of Risk score, gender, age, pathologic stage, and histologic grade by using TCGA-BLCA data. **(D)** Forest plot summary of analyses of DFS multivariate analysis of Risk score, age, and pathologic stage by using TCGA-BLCA data.

According to the results of the univariable Cox analysis, risk score (*P* = 0.001), age (*P* < 0.001), and pathologic stage (*P* < 0.001) are significant risk features for OS (**Figure 4A**). Even when adjusted by other clinical features, the risk score was significantly associated with the OS of BC, which could become an independent factor for prognosis prediction, suggested by multivariate Cox analysis (**Figure 4B**). We also conducted a univariable Cox analysis by setting DFS as the endpoint, the result indicated that risk score (*P* = 0.006) and pathologic stage (*P* = 0.002) were risk features for DFS, significantly (**Figure 4C**). Unfortunately, subsequent multivariable Cox analysis demonstrated that the risk score (*P* = 0.099) might not act as an independent factor for DFS prediction. As indicated above, this autophagy-related signature showed better potential for OS prediction compared to DFS prediction (**Figure 4D**).

### Prognostic Value of the Risk Signature Exploration Stratified by Clinicopathological Features

**Figure 5:**
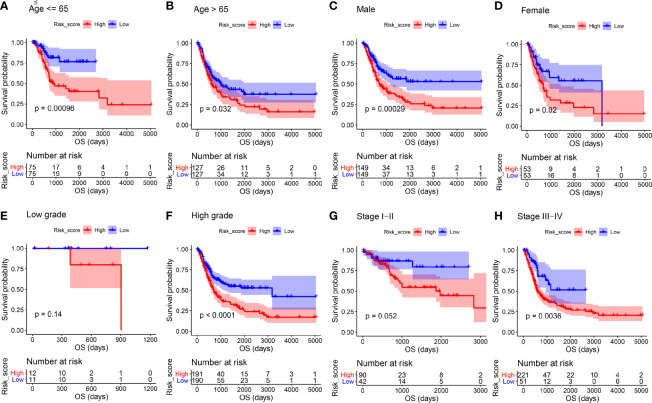
Kaplan-Meier overall survival curves for the high- and low-risk groups stratified by clinicopathological variables. **(A, B)** Age. **(C, D)** Gender. **(E, F)** Grade. **(G, H)** Stage.

We then stratified BC patients using age, gender, histologic grade, and pathologic stage to assess the prognostic value of the ARG-based signature for OS of patients with BC. In age ≤ 65 (**Figure 5A**, *P* < 0.001), age > 65 (**Figure 5B**, *P* = 0.032), male (**Figure 5C**, *P* < 0.001), female (**Figure 5D**, *P* = 0.020), high histologic grade (**Figure 5F**, *P* < 0.001), and stage III-IV subgroups (**Figure 5H**, *P* = 0.004), high-risk group patients with BC had obviously worse OS, as the survival analyses suggested. In low histologic grade (**Figure 5E**, *P* = 0.140), and stage I-II subgroups (**Figure 5G**, *P* = 0.052), there was a trend that BC patients in the low-risk group had higher OS time when compared with the high-risk group. We have investigated these results and concluded that the risk signature for OS could act as an independent prognosis prediction tool of BC patients without thinking about clinical features.

We then assessed the prognostic value of the ARG-based signature for the DFS of patients. Similarly, in age ≤ 65 (**Figure S8A**, *P* = 0.035), age > 65 (**Figure S8B**, *P* = 0.004), male (**Figure S8C**, *P* < 0.001), high histologic grade (**Figure S8F**, *P* < 0.001), stage I-II (**Figure S8G**, *P* = 0.045), and stage III-IV subgroups (**Figure S8H**, *P* = 0.011), BC patients in the high-risk group occupied worse DFS compared to those in the low-risk group. In subgroups of female patients (**Figure S8D**, *P* = 0.690) and those with low histologic grade (**Figure S8E**, *P* = 0.170) there was a trend that BC patients in the low-risk group occupied better DFS compared to those in the high-risk group. The above results indicated that the risk signature might play an independent role in predicting the DFS of BC patients.

### The ARG-Based Model for OS Could Predict the Progression of BC

**Figure 6:**
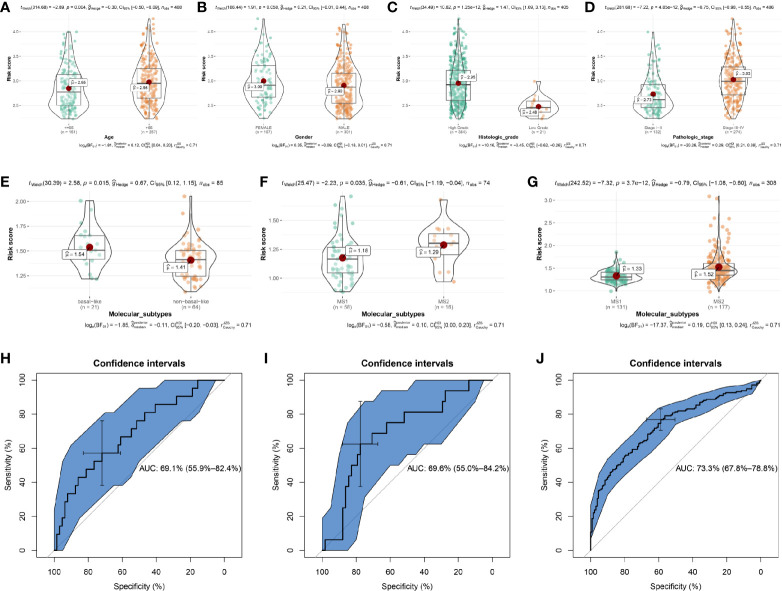
The relationships between the risk score and clinicopathological variables and the prediction value of risk score to molecular subtypes. The relationships between the risk score and **(A)** Age. **(B)** Gender. **(C)** Histologic grade. **(D)** Pathologic stage. The relationships between the risk score and molecular subtypes (non-basal-like/MS1 *vs* basal-like/MS2) by using E-MTAB-1803 **(E)**, GSE19915 **(F)**, and GSE32894 **(G)**. Receiver operating characteristic (ROC) curves and area under the curve (AUC) statistics to evaluate the diagnostic efficiency of risk score to bladder cancer aggressiveness in E-MTAB-1803 **(H)**, GSE19915 **(I)**, and GSE32894 **(J)**.

The association between the risk score signature and clinical features was then explored to examine whether the signature was related to the progression of BC. The risk scores of patients in the age ≤ 65 group were lower than those in the age > 65 group (**Figure 6A**, *P* = 0.004). There was a trend that female patients occupied higher risk scores than male patients (**Figure 6B**, *P* = 0.058). Moreover, the risk scores of BC patients in the high histologic grade were significantly higher than those in the low histologic group (**Figure 6C**, *P* = 1.25e-12). In addition, **Figure 6D** suggests that the risk scores of BC patients in the stage I-II group were lower than those in the stage III-IV group, accurately (*P* = 4.85e-12). According to these results, the progression of patients with BC was related to this risk signature for OS.

### ARG-Based Model for OS Associated With the Molecular Subtypes of BC

To test whether the signature was related to the molecular subtypes of BC, we then examined the association between the risk score signature and molecular subtypes. Based on E-MTAB-1803, we first calculated the risk scores for the 85 BC samples, showing in **Table S11**. Combined with the molecular subtype information for each sample, we found that BC patients with basal-like subtype had higher risk score levels compared to those with non-basal-like subtype (**Figure 6E**, *P* = 0.015). It is known that the basal subtype was associated with more aggressive cancers, thus, the result indicated that the risk score level was positively associated with bladder cancer invasiveness. Lindgren et al. refined the classification of BC in their studies (19, 22) and identified two intrinsic molecular subtypes, MS1 and MS2. This study proved that the BCs of the MS2 subtype were strongly associated with aggressive growth and poor prognosis. In exploring whether the risk score level was positively related to BC invasiveness, we used the subtype information of GSE19915 (19) and GSE32894 (22) to validate the relationship. The results demonstrated that BC patients with MS2 subtype had higher risk scores compared to patients with MS1 subtype, by using GSE19915 (**Figure 6F**, *P* = 0.035) and GSE32894 (**Figure 6G**, *P* = 3.7e-12).

We further explored the prediction value of risk score to BC aggressiveness. We found that the risk score could distinguish more aggressive BCs (MS2/basal subtype) among BCs well (E-MTAB-1803, **Figure 6H**, AUC = 0.691; GSE19915, **Figure 6I**, AUC = 0.696; GSE32894, **Figure 6J**, AUC = 0.733). Taken together, the risk score calculated by the ARG-based model was positively associated with bladder cancer invasiveness, which could predict aggressive cancer features.

### The Prognostication Value of the ARG-Based Signature to Disease Stage and Tumor Grade

To explore the prognostication value of the ARG-based signature to disease stage and tumor grade, we performed ROC analysis using E-MTAB-4321, GSE13507, GSE19915, GSE32894, and TCGA-BLCA data. The ARG-based signature could distinguish Ta-T1 stage BCs from T2-T4 stage BCs well, by using the E-MTAB-4321 cohort (**Figure S9A**, AUC = 0.782). By using dataset GSE13507, we also demonstrated that there was a trend that the autophagy-related signature showed good potential in distinguishing Ta-T1 stage BCs and T2-T4 stage BCs (**Figure S9B**, AUC = 0.726). Subsequent analysis based on the GSE19915 cohort indicated that the ARG-based signature showed strong potential in distinguishing the BCs of the Ta-T1 stage and BCs of the T2-T4 stage (**Figure S9C**, AUC = 0.897). By using GSE32894, we reached a similar conclusion (**Figure S9D**, AUC = 0.705) and immediately explored the prognostication value of the ARG-based signature to pathologic stage by using TCGA-BLCA data, the result suggested that there was a trend that the ARG-based signature could distinguish the BCs of stage I-II from the BCs of stage III-IV (**Figure S9E**, AUC = 0.719). Subsequent analysis indicated that the ARG-based signature might not play a part in distinguishing high grade and low grade BCs using E-MTAB-4321(**Figure S9F**, AUC = 0.555), GSE13507 (**Figure S9G**, AUC = 0.666), and GSE32894 (**Figure S9I**, AUC = 0.621). However, by using GSE19915 (**Figure S9H**, AUC = 0.741) and TCGA-BLCA data (**Figure S9J**, AUC = 0.851), the ARG-based signature could distinguish BCs of high grade from BCs of low grade well.

We found that the ARG-based signature could distinguish BCs of Ta-T1 stage from BCs of T2-T4 stage well, which indicated that this signature has prognostication value at the disease stage. As for the prognostication value of the ARG-based signature to tumor grade, the results were inconsistent when we used different datasets, which suggests that the prediction value of the ARG-based signature was unsteady.

### Nomograms With and Without Autophagy-Related Signature Based on Clinical Utility Were Constructed

**Figure 7:**
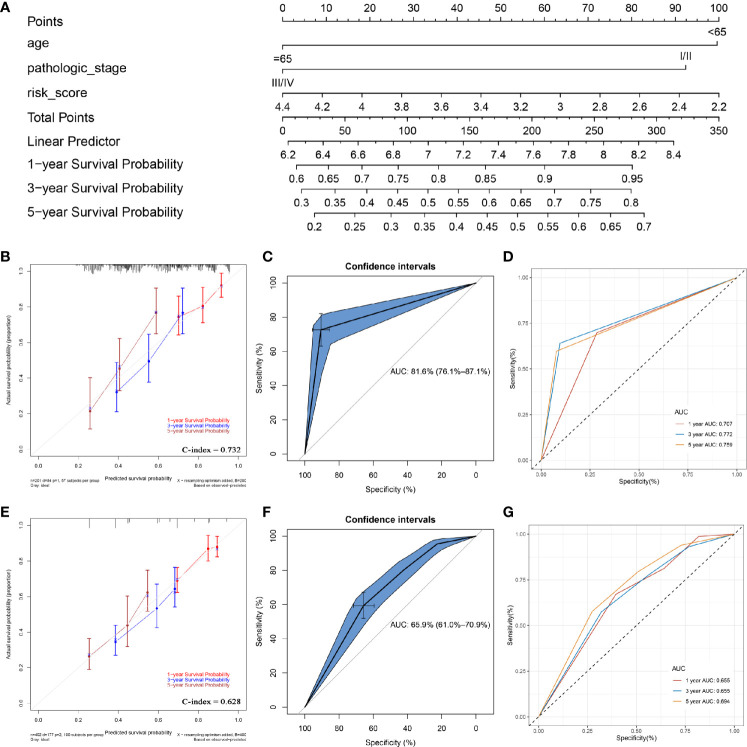
Construction of a nomogram with/without the ARG signature. **(A)** the nomogram with the ARG signature for predicting the proportion of patients with 1-, 3- or 5-year OS. **(B)** the calibration plots for predicting 1-, 3- or 5- year OS by using TCGA-BLCA data. **(C)** receiver operating characteristic (ROC) curves and area under the curve (AUC) statistics to evaluate the diagnostic efficiency of the nomogram with ARG signature in TCGA-BLCA data. **(D)** Time-dependent ROC curves indicating the predictive accuracy of the nomogram with ARG signature for 1-, 3-, or 5- year OS. **(E)** the calibration plots for predicting 1-, 3- or 5- year OS by using TCGA-BLCA data. **(F)** receiver operating characteristic (ROC) curves and area under the curve (AUC) statistics to evaluate the diagnostic efficiency of the nomogram without ARG signature in TCGA-BLCA data. **(G)** Time-dependent ROC curves indicating the predictive accuracy of the nomogram without ARG signature for 1-, 3-, or 5- year OS.

In order to apply this prognostic signature in clinical work, a nomogram was constructed based on a risk score assessed by the prognostic signature, age, and pathologic stage (independent factors verified by multivariate Cox analysis) (**Figure 7A**). According to the calibrate curve, this nomogram showed good performance in survival probability prediction, especially for long term survival rate (5-year OS, **Figure 7B**). By conducting ROC analysis, we demonstrated that the nomogram could predict the OS of BC patients effectively (C-index: 0.732; AUC: 0.816; **Figure 7C**). Time-dependent ROC curves demonstrated that this nomogram with ARG signature showed excellent stability over a period of 5 years (1-year AUC: 0.707, 3-years AUC: 0.772, 5-years AUC: 0.759, **Figure 7D**). This nomogram expressed better potential for long term survival rate prediction (3- and 5- year OS).

To prove the prediction power of the prognostic signature (risk score), we also established another nomogram, assessed only by the age and pathologic stage (**Figure S10**) without the ARG signature. This nomogram did not show the same performance as the ARG-signature-based nomogram (**Figure 7E**). In addition, as shown in **Figure 7F**, both the C-index (0.628) and AUC value (0.659) for the nomogram without ARG signature were lower than these of nomogram with ARG signature, which proved that the ARG signature improved the clinical net benefit of the standard model based on clinicopathological features (age, pathologic stage). The time-dependent ROC curves also demonstrated that the predictive value of the nomogram without ARG signature (1-year AUC: 0.655, 3-years AUC: 0.655, 5-years AUC: 0.694) did not match the nomogram with ARG signature (**Figure 7G**).

### Comparison of Nomograms With/Without ARG Signature to Prove the Predictive Value of the ARG Signature

**Figure 8:**
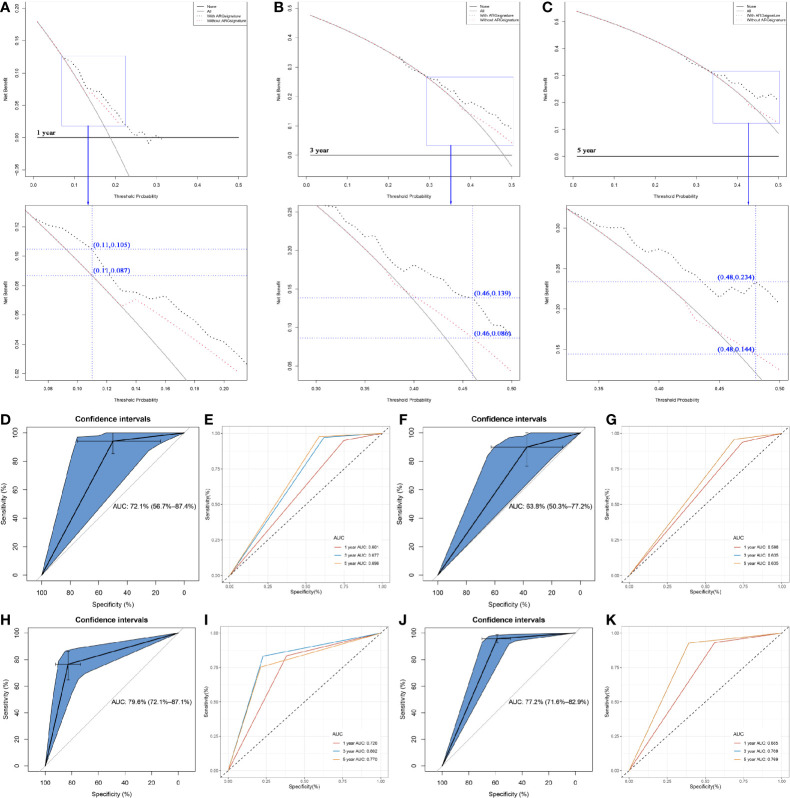
Comparison of the nomogram with/without the ARG signature. **(A)** Decision curve analysis (DCA) for assessment of the clinical utility for 1- year OS of the nomogram with/without the ARG signature. **(B)** Decision curve analysis (DCA) for assessment of the clinical utility for 3- year OS of the nomogram with/without the ARG signature. **(C)** Decision curve analysis (DCA) for assessment of the clinical utility for 5- year OS of the nomogram with/without the ARG signature. **(D)** Receiver operating characteristic (ROC) curves and area under the curve (AUC) statistics to evaluate the diagnostic efficiency of the nomogram with ARG signature in GSE31684. **(E)** Time-dependent ROC curves indicating the predictive accuracy of the nomogram with ARG signature for 1-, 3-, or 5- year OS based on GSE31684. **(F)** Receiver operating characteristic (ROC) curves and area under the curve (AUC) statistics to evaluate the diagnostic efficiency of the nomogram without ARG signature in GSE31684. **(G)** Time-dependent ROC curves indicating the predictive accuracy of the nomogram without ARG signature for 1-, 3-, or 5- year OS based on GSE31684. **(H)** Receiver operating characteristic (ROC) curves and area under the curve (AUC) statistics to evaluate the diagnostic efficiency of the nomogram with ARG signature in GSE13507. **(I)** Time-dependent ROC curves indicating the predictive accuracy of the nomogram with ARG signature for 1-, 3-, or 5- year OS based on GSE13507. **(J)** Receiver operating characteristic (ROC) curves and area under the curve (AUC) statistics to evaluate the diagnostic efficiency of the nomogram with ARG signature in IMvigor210. **(K)** Time-dependent ROC curves indicating the predictive accuracy of the nomogram with ARG signature for 1-, 3-, or 5- year OS based on IMvigor210.

DCA was performed to evaluate the clinical net benefit of using both the nomogram with and without ARG signature in predicting the probability of 1-, 3-, and 5- year survival. As **Figure 8A** shows, the two models had nearly identical decision curves when threshold probability was (*Pt*) < 0.08, which meant the nomogram with ARG signature failed to improve the net-benefit for 1-year survival prediction compared to the nomogram without ARG signature.

Subsequent analysis indicated that there was a trend that the nomogram with ARG signature had a higher net benefit than the nomogram without ARG signature when *Pt* ranged between 0.08 to 0.21. At the 0.11 *Pt*, the net benefit was 0.105 in the nomogram with ARG signature and 0.087 in the nomogram without ARG signature (**Figure 8A**). The max improved net benefit was approximately 0.018, which might not be significant. For the evaluation of the clinical net benefit using the nomogram with ARG for predicting 3-year survival probability, the nomogram with ARG model had a higher net benefit compared to the nomogram without ARG model, especially when *Pt* ranged from 0.29 to 0.50 (**Figure 8B**). When *Pt* was 0.46, the net benefit was improved from 0.086 to 0.139 (**Figure 8B**). The nomogram with ARG signature also obviously improved the net benefit for 5-year survival prediction compared to the nomogram without ARG signature. The nomogram with ARG signature had a higher net benefit than the simple nomogram of *Pt* between 0.33 and 0.50 (**Figure 8C**). When *Pt* was 0.48, the net benefit was improved from 0.144 to 0.234 (**Figure 8C**). The nomogram with ARG signature shows high potential for clinical applications, especially for 3- and 5- year survival prediction.

We also validated the prediction value of the two nomograms by using GSE31684. The nomogram with ARG signature could predict the OS of BC patients effectively (AUC: 0.721; **Figure 8D**). Time-dependent ROC curves demonstrated that this nomogram with ARG signature showed good stability over a period of 5 years (1-year AUC: 0.601, 3-years AUC: 0.677, 5-years AUC: 0.696, **Figure 8E**). As **Figure 8F** (AUC: 0.638) and **Figure 8G** (1-year AUC: 0.598, 3-years AUC: 0.635, 5-years AUC: 0.635) show, we reached a similar conclusion, that the nomogram with ARG signature showed a better predictive value compared to the nomogram without ARG signature, which demonstrated that the ARG signature improved the clinical net benefit of the standard model based on clinicopathological features (age and pathologic stage).

Two independent datasets GSE13507 and IMvigor210 with complete OS information were used to validate the prediction value of the nomogram with ARG signature. The results suggested that the nomogram with ARG signature showed strong potential in the prediction of BC patients’ OS (GSE13507: **Figure 8H**, AUC = 0.796; IMvigor210: **Figure 8J**, AUC = 0.772). Time-dependent ROC curves demonstrated that the nomogram with ARG signature showed good stability over a period of 5 years by using GSE13507 (1-year AUC: 0.726, 3-years AUC: 0.802, 5-years AUC: 0.770, **Figure 8I**) and IMvigor210 (1-year AUC: 0.685, 3-years AUC: 0.769, 5-years AUC: 0.769, **Figure 8K**). The nomogram with ARG signature had strong potential in the prediction of BC patients’ OS.

### Identifying the Risk Signature Associated With KEGG Signaling Pathways

GSEA was performed to explore the potential roles of the risk signature. Using the cut-off criteria that had been set previously, the risk score assessed by this prognostic signature was significantly associated with cell adhesion molecules cams, focal adhesion, leukocyte transendothelial migration, vascular smooth muscle contraction, regulation of actin cytoskeleton, neuroactive ligand receptor interaction, calcium signaling pathway, chemokine signaling pathway, cytokine cytokine receptor interaction, and toll like receptor signaling pathway (**Table S12**).

## Discussion

Bladder cancer (BC) is a malignant tumor that endangers human health. It occurs on the bladder mucosa (1). Methods such as TNM staging have been widely used to predict the prognosis of patients with BC (40). However, unfortunately, TNM staging does not always function well (6) and there is a great need for more effective prognostic risk models. Moreover, it has been reported that autophagy is closely associated with the prognosis of tumor patients. The present study thus aimed to develop an autophagy-related prognostic signature.

A co-expression network was constructed using TCGA-BLCA, and two modules including a brown module and a blue module were identified and considered to be related to the disease. 123 ARGs from the disease-related modules were selected for subsequent analysis. 70 differentially expressed ARGs were immediately screened out with the standards we set. The genes that overlapped between the 123 ARGs and the 70 ARGs were immediately picked out, as they are mainly enriched in some autophagy-related signaling pathways.

Survival analysis was subsequently performed to screen out potential prognostic biomarkers. We selected nine ARGs that showed significant P values in both OS analysis and DFS analysis. The expression levels of all the nine ARGs in BCs and normal tissues were verified with significant differences. Univariate and multivariate Cox analyses were then conducted to screen out genes from the potential prognostic biomarkers to construct an autophagy-related prognostic signature. Three genes including BOC, FGF7, and MAP1A were selected and included for the risk signature construction.

After a literature search, we found that no studies explained the roles of BOC and FGF7 in BC. BOC was reported to be overexpressed in patients with glioblastoma multiforme and related to poor survival outcomes (41). Hong et al. concluded that BOC was a modifier gene in holoprosencephaly (42). The FGF7 signaling was reported to be disrupted in colorectal cancer, which could be a potential marker of field cancerization (43). Moreover, Zho et al. found that MiR-199a-3p could regulate FGF7 and further inhibit the proliferation, migration, and invasion of endothelial cells and pericytes in diabetic retinopathy rats (44). MAP1A is a member of the microtubule-associated protein family that is involved in microtubule assembly (an essential step in neurogenesis). Song et al. demonstrated that MAP1A was significantly overexpressed in pancreatic ductal adenocarcinoma (45). MAP1A was also reported to be a prognostic biomarker in prostate cancer (46). Based upon these previous findings, we concluded that these three ARGs might be novel prognostic biomarkers in BC.

Autophagy is a controversial cellular process because it can show both tumor suppressor and oncogenic functions (9, 10). This process contains three main steps: initiation, elongation, and maturation (10). Recently, some studies have demonstrated that ARGs play crucial roles in the development of BC. Wang et al. developed an individualized autophagy-clinical prognostic index that could robustly estimate the survival of BC patients (12). Eissa et al. identified four autophagy transcripts regulating the three main steps of autophagy, a novel panel for diagnosis of BC (47). These results indicate that autophagy is essential for BC and ARGs might act as prognostic or diagnostic biomarkers for BC.

Our study indicated that overexpression of BOC, FGF7, and MAP1A was significantly correlated to an advanced pathological stage and high grade. Moreover, the high expression of these ARGs caused inferior OS. Based upon the above, we hypothesized that BOC, FGF7, and MAP1A might act as major driving forces in bladder cancer progression. However, it is hard to say which autophagy process the three ARGs majorly affect. Subsequent GSEA demonstrated that the ARG-based signature assessed by BOC, FGF7, and MAP1A was significantly enriched in cell adhesion molecules cams, focal adhesion, leukocyte transendothelial migration, vascular smooth muscle contraction, regulation of actin cytoskeleton, neuroactive ligand receptor interaction, calcium signaling pathway, chemokine signaling pathway, cytokine-cytokine receptor interaction, and toll like receptor signaling pathway. Hence, we speculate that BOC, FGF7, and MAP1A might influence the autophagy process through regulating and controlling these signaling pathways, and thereby affect the prognosis of BC. More in-depth studies must be conducted to understand the mechanism better in the near future.

To the best of our knowledge, the autophagy-related signature outlined in the present study is the first to be constructed for the prediction of the prognosis of BC patients. This risk signature was validated using an external dataset. By conducting Cox proportional hazards regression analysis, this ARG-based signature was determined to be an independent prognostic feature for patients with BC, which meant that we could predict the prognosis of patients through this risk signature without considering other clinical features. Subsequent analysis indicated that the risk score calculated by the ARG-based model was positively associated with bladder cancer invasiveness, which could predict aggressive cancer features.

In order to make the risk signature a clinical reality, we established two nomograms with and without an autophagy-related signature, based on age and pathologic stage. Subsequent analysis indicated that the nomogram without ARG signature did not show the same performance as the ARG-signature-based nomogram. These results proved that the ARG signature improved the clinical net benefit of the standard model based on clinicopathological features (age, pathologic stage). The ARG-signature-based nomogram was subsequently validated and showed high accuracy in predicting the probability of overall survival for patients with BC.

There are some limitations to the present study. First, the roles of the three ARGs need to be validated using *in vivo* and *in vitro* experiments. Second, when we explored the prognostication value of the ARG-based signature to tumor grade, the results were inconsistent by using different datasets. Some exploration for this problem must be conducted using our own data in the future. Third, although we conducted internal and external validation for this prognostic signature, there was a lack of validation using our own data. This study will be developed in the future by collecting data in a clinical setting and undertaking a prospective clinical trial.

## Conclusion

The present study combined WGCNA and DEG identification to explore potential prognostic ARGs in BC. To the best of our knowledge, this was the first time that an autophagy-related prognostic signature was constructed based on ARGs in BC. This signature was positively associated with bladder cancer invasiveness and could act as an effective prediction tool for the prognosis of BC patients independently. An ARG-signature-based nomogram was established based on the risk score assessed by this risk signature to provide clinical doctors with a visual tool, meaning the probability of BC patient survival could be more precisely predicted.

## Data Availability Statement

The datasets presented in this study can be found in online repositories. The names of the repository/repositories and accession number(s) can be found in the article/**Supplementary Material**.

## Author Contributions

T-ZL and XY conceived and designed the study. XY and ZC performed the analysis procedures. G-WD, XY, ZC, KT, X-JB, H-HW, and T-ZL analyzed the outcomes. T-ZL and XY contributed analysis tools. XY and ZC contributed to the writing of the manuscript. XY and H-HW revised the manuscript. All authors contributed to the article and approved the submitted version.

## Funding

The present study was supported by the Medical science and technology innovation platform support project of Zhongnan Hospital of Wuhan University (PTXM2019006).

## Conflict of Interest

The authors declare that the research was conducted in the absence of any commercial or financial relationships that could be construed as a potential conflict of interest.

## Supplementary Material

Supplementary Figure 1 The flow diagram of this study. Data preparation, analysis, and validation are shown in the flow diagram.

Supplementary Figure 2
**(A)** Sample clustering to detect outliers. **(B)** Sample dendrogram and trait heatmap. The color intensity was proportional to disease status.

Supplementary Figure 3 Determination of soft-thresholding power in the weighted gene co-expression network analysis (WGCNA). **(A)** Analysis of the scale-free fit index for various soft-thresholding powers (β). **(B)** Analysis of the mean connectivity for various soft-thresholding powers. **(C)** Histogram of connectivity distribution when β = 4. **(D)** Checking the scale free topology when β = 4.

Supplementary Figure 4 Identification of potential prognostic biomarkers. **(A)** Identification of overlapped ARGs between hub genes from WGCNA and DEGs. **(B)** GO biological processes analysis. **(C)** KEGG pathway enrichment.

Supplementary Figure 5 Kaplan–Meier disease free survival (DFS) curves for BLCA patients assigned to groups of high and low expression level of based on the nine genes, respectively. (A–I show the results of ABL1, BOC, EIF4EBP1, FGF7, KCNK3, MAP1A, MYLK, PPARGC1A, REEP2, respectively).

Supplementary Figure 6 Visualization of the expression levels of the 9 differentially expressed autophagy-related genes based on TCGA-BLCA data **(A)** and GSE13507 **(B)**. Red represents bladder cancer tissue, and blue represents normal tissue.

Supplementary Figure 7 The correlation between the three-gene autophagy-related signature and the OS, CSS, PFS, and DFS of patients with BC. **(A)** Kaplan-Meier OS curves for the high- and low-risk groups by using IMvigor210. **(B)** ROC curve indicating the predictive accuracy of the autophagy-related signature for OS by using IMvigor210. **(C)** Kaplan-Meier OS curves for the high- and low-risk groups by using GSE13507. **(D)** ROC curve indicating the predictive accuracy of the autophagy-related signature for OS by using GSE13507. **(E)** Kaplan-Meier CSS curves for the high- and low-risk groups by using GSE13507. **(F)** ROC curve indicating the predictive accuracy of the autophagy-related signature for CSS by using GSE13507. **(G)** Kaplan-Meier CSS curves for the high- and low-risk groups by using GSE19915. **(H)** ROC curve indicating the predictive accuracy of the autophagy-related signature for CSS by using GSE19915. **(I)** Kaplan-Meier CSS curves for the high- and low-risk groups by using GSE31684. **(J)** ROC curve indicating the predictive accuracy of the autophagy-related signature for CSS by using GSE31684. **(K)** Kaplan-Meier CSS curves for the high- and low-risk groups by using GSE32894. **(L)** ROC curve indicating the predictive accuracy of the autophagy-related signature for CSS by using GSE32894. **(M)** Kaplan-Meier PFS curves for the high- and low-risk groups by using GSE19915. **(N)** ROC curve indicating the predictive accuracy of the autophagy-related signature for PFS by using GSE19915. **(O)** Kaplan-Meier PFS curves for the high- and low-risk groups by using GSE32894. **(P)** ROC curve indicating the predictive accuracy of the autophagy-related signature for PFS by using GSE32894. **(Q)** Kaplan-Meier PFS curves for the high- and low-risk groups by using E-MTAB-4321. **(R)** ROC curve indicating the predictive accuracy of the autophagy-related signature for PFS by using E-MTAB-4321. **(S)** Kaplan-Meier DFS curves for the high- and low-risk groups by using TCGA-BLCA data. **(T)** ROC curve indicating the predictive accuracy of the autophagy-related signature for DFS by using TCGA-BLCA data.

Supplementary Figure 8 Kaplan-Meier disease-free survival curves for the high- and low-risk groups stratified by clinicopathological variables. **(A, B)** Age. **(C, D)** Gender. **(E, F)** Grade. **(G, H)** Stage.

Supplementary Figure 9 The prognostication value of the ARG-based signature to disease stage and tumor grade by ROC curves. ROC curves to distinguish BCs of Ta-T1 stage from BCs of T2-T4 stage by using E-MTAB-4321 **(A)**, GSE13507 **(B)**, GSE19915 **(C)**, GSE32894 **(D)**. ROC curves to distinguish BCs of Ta-T1 stage from BCs of T2-T4 stage by using TCGA-BLCA data **(E)**. ROC curves to distinguish BCs of high grade from BCs of low grade by using E-MTAB-4321 **(F)**, GSE13507 **(G)**, GSE19915 **(H)**, GSE32894 **(I)**, and TCGA-BLCA data **(J)**.

Supplementary Figure 10 The nomogram without the ARG signature for predicting the proportion of patients with 1-, 3- or 5-year OS.
