# Supplementary figures and images for: Construction and Validation of an Autophagy-Related Prognostic Signature and a Nomogram for Bladder Cancer
Source: Front Oncol. 2021 Jun 18;11:632387. doi: 10.3389/fonc.2021.632387 (PMC8252967; doi:10.3389/fonc.2021.632387)

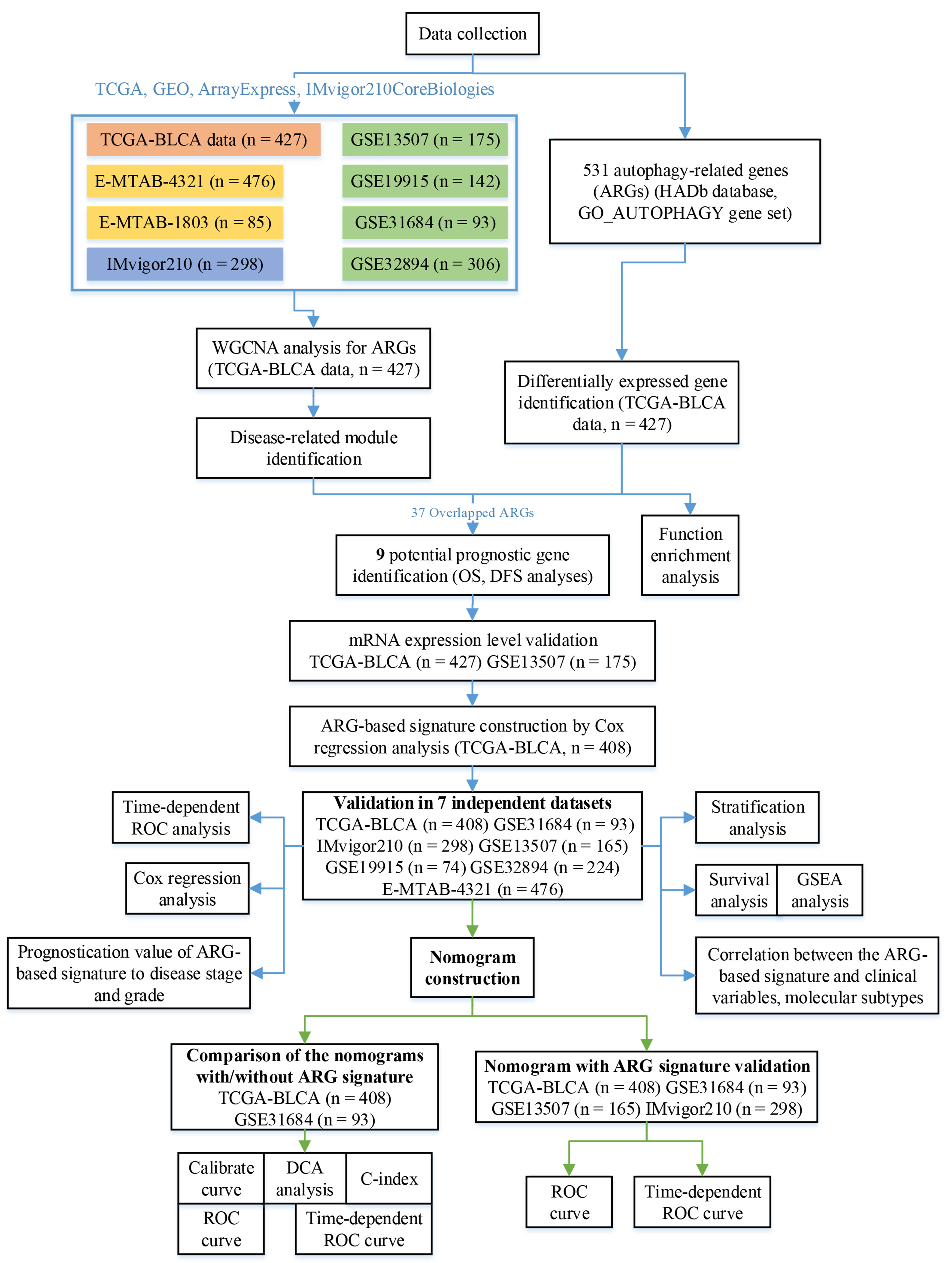

Supplement: Supplementary Figure 1 — The flow diagram of this study. Data preparation, analysis, and validation are shown in the flow diagram. [file Image_1.tif]

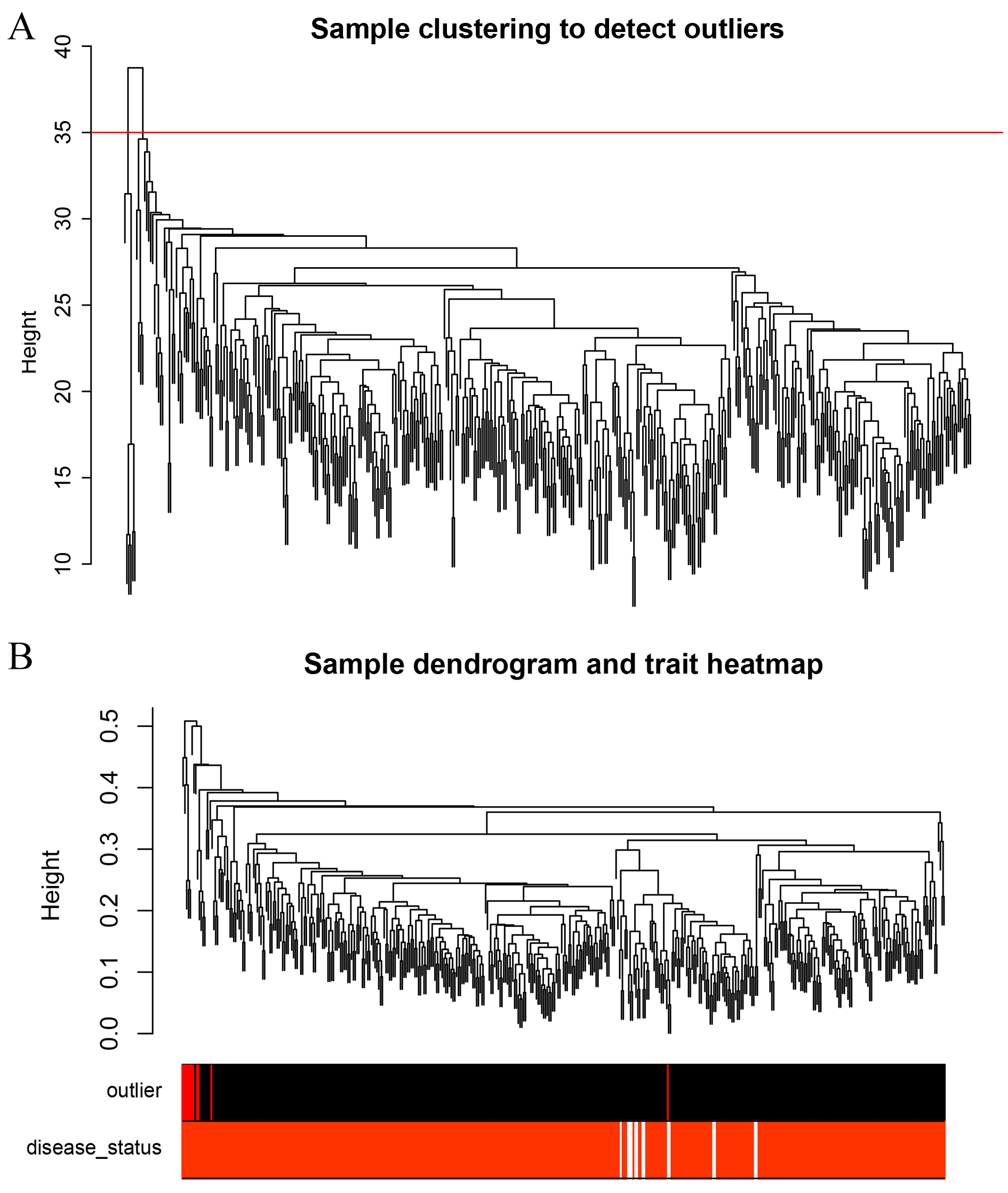

Supplement: Supplementary Figure 2 — (A) Sample clustering to detect outliers. (B) Sample dendrogram and trait heatmap. The color intensity was proportional to disease status. [file Image_2.tif]

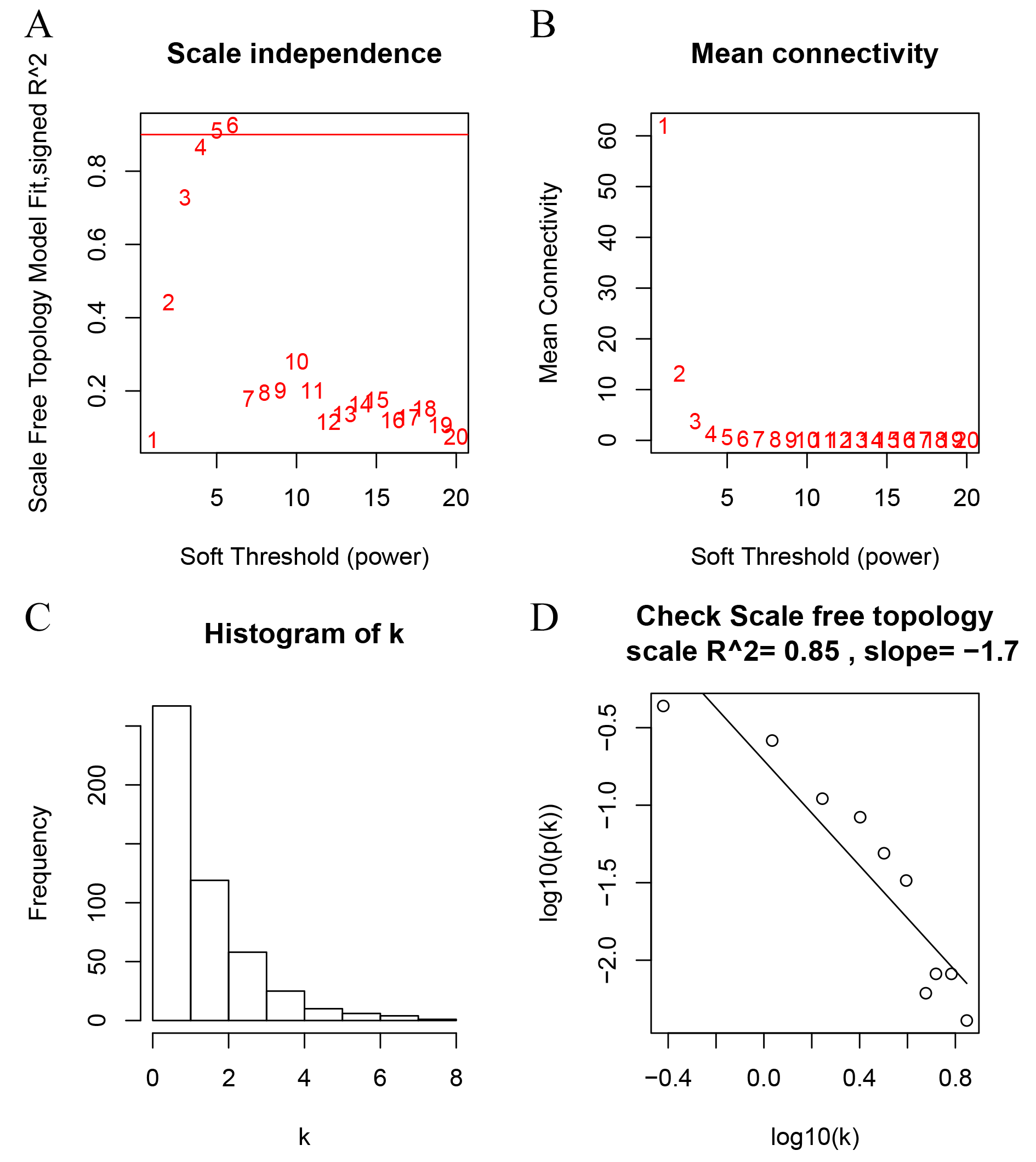

Supplement: Supplementary Figure 3 — Determination of soft-thresholding power in the weighted gene co-expression network analysis (WGCNA). (A) Analysis of the scale-free fit index for various soft-thresholding powers (β). (B) Analysis of the mean connectivity for various soft-thresholding powers. (C) Histogram of connectivity distribution when β = 4. (D) Checking the scale free topology when β = 4. [file Image_3.tif]

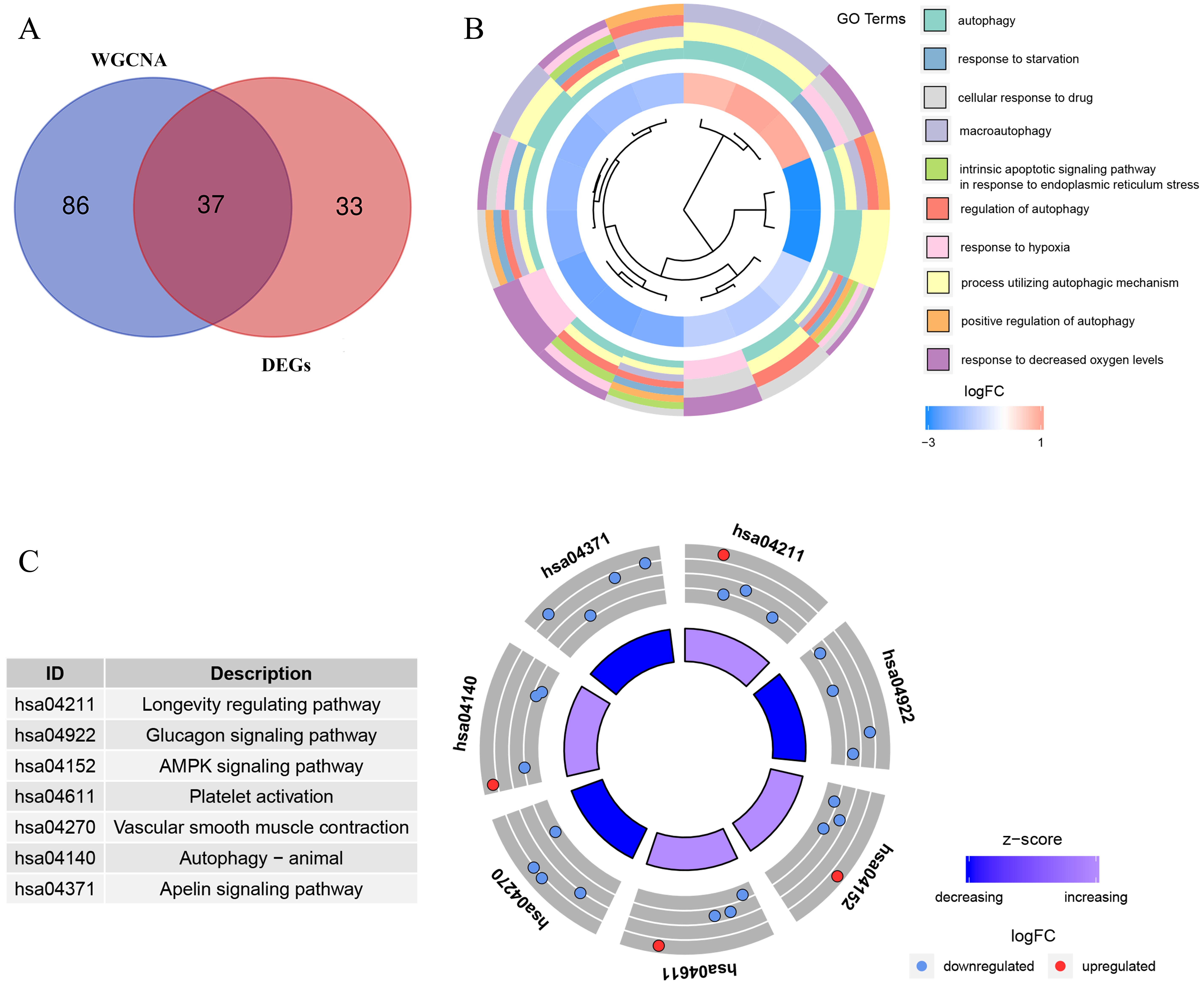

Supplement: Supplementary Figure 4 — Identification of potential prognostic biomarkers. (A) Identification of overlapped ARGs between hub genes from WGCNA and DEGs. (B) GO biological processes analysis. (C) KEGG pathway enrichment. [file Image_4.tif]

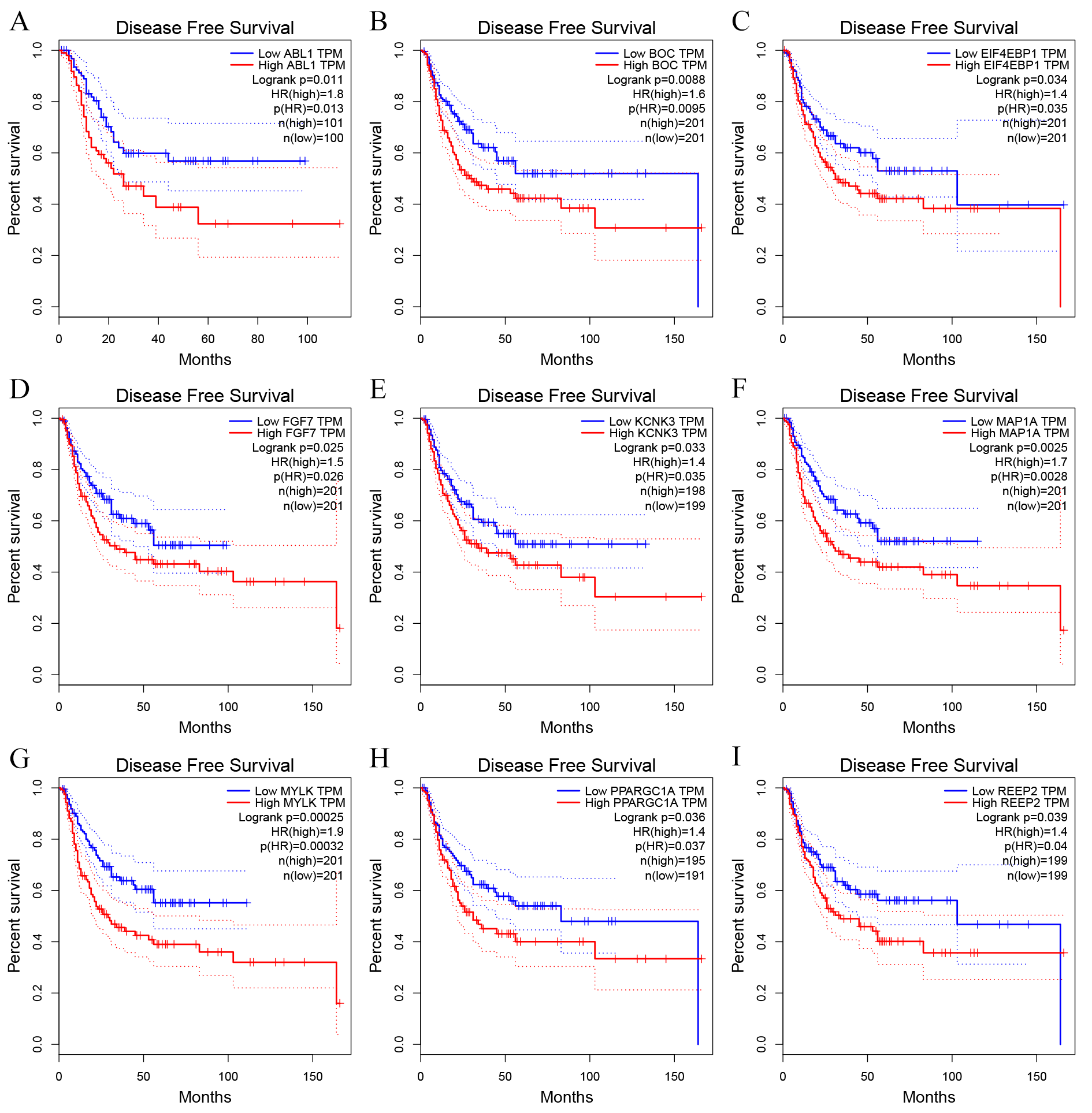

Supplement: Supplementary Figure 5 — Kaplan–Meier disease free survival (DFS) curves for BLCA patients assigned to groups of high and low expression level of based on the nine genes, respectively. (A–I show the results of ABL1, BOC, EIF4EBP1, FGF7, KCNK3, MAP1A, MYLK, PPARGC1A, REEP2, respectively). [file Image_5.tif]

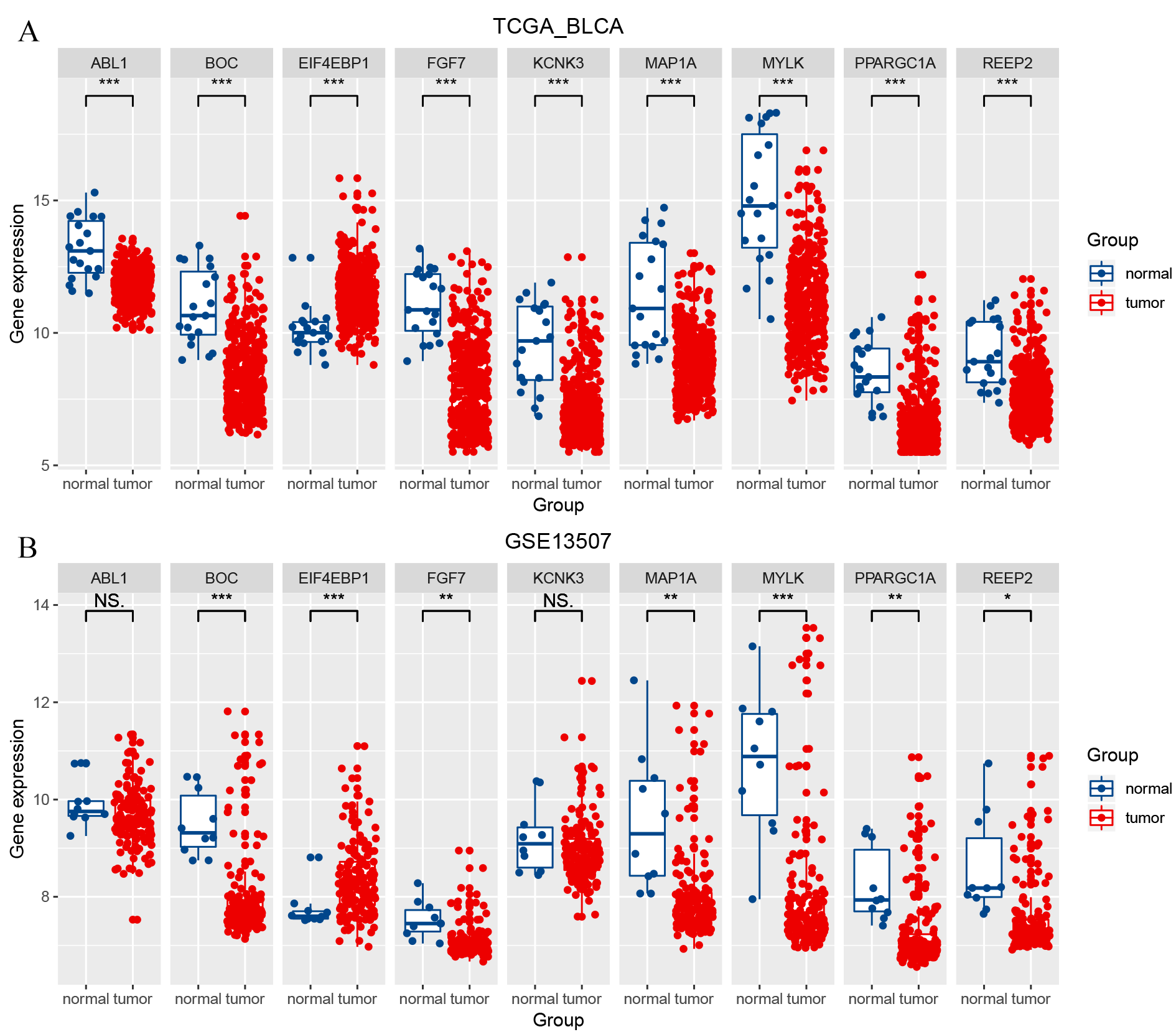

Supplement: Supplementary Figure 6 — Visualization of the expression levels of the 9 differentially expressed autophagy-related genes based on TCGA-BLCA data (A) and GSE13507 (B). Red represents bladder cancer tissue, and blue represents normal tissue. [file Image_6.tif]

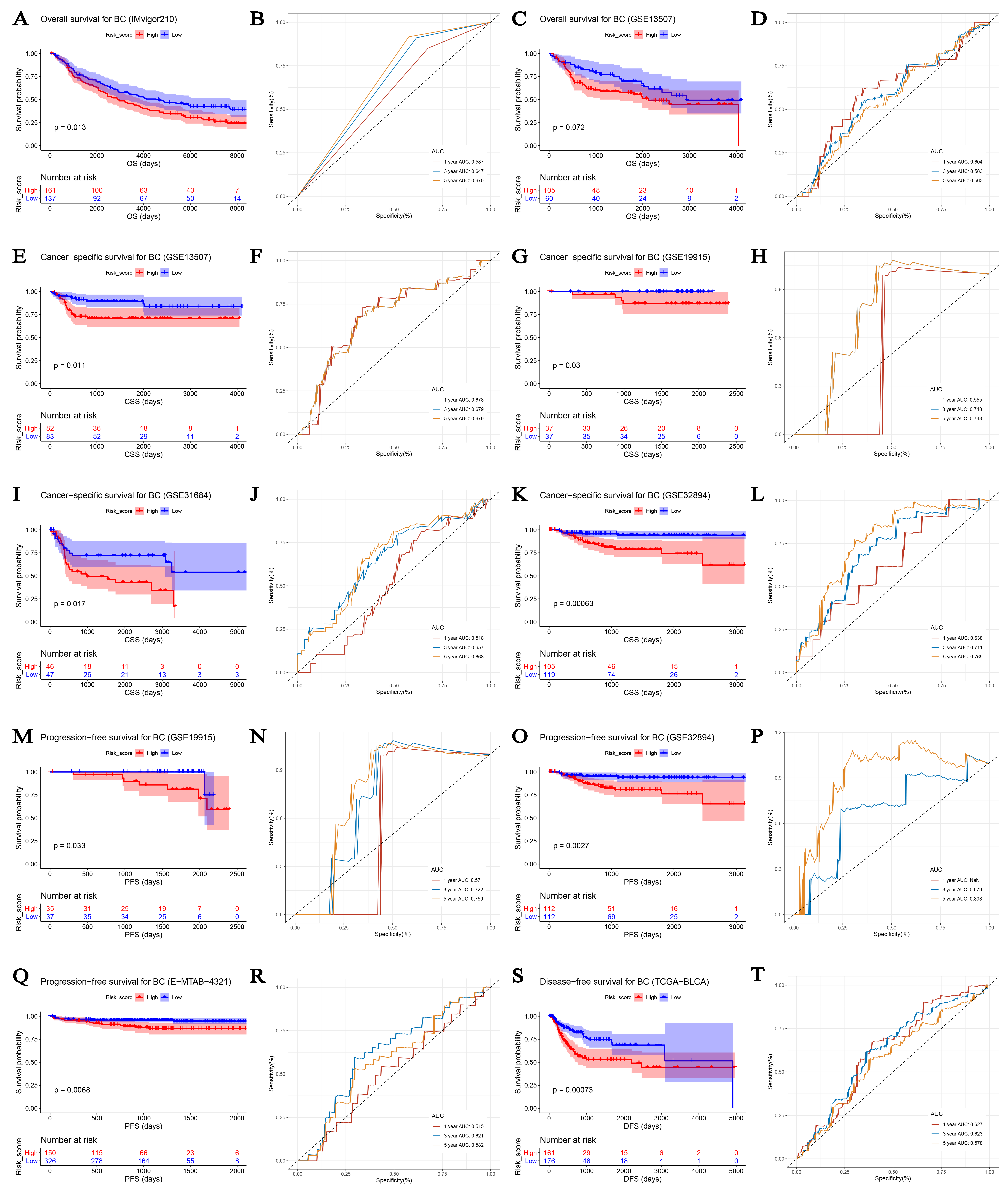

Supplement: Supplementary Figure 7 — The correlation between the three-gene autophagy-related signature and the OS, CSS, PFS, and DFS of patients with BC. (A) Kaplan-Meier OS curves for the high- and low-risk groups by using IMvigor210. (B) ROC curve indicating the predictive accuracy of the autophagy-related signature for OS by using IMvigor210. (C) Kaplan-Meier OS curves for the high- and low-risk groups by using GSE13507. (D) ROC curve indicating the predictive accuracy of the autophagy-related signature for OS by using GSE13507. (E) Kaplan-Meier CSS curves for the high- and low-risk groups by using GSE13507. (F) ROC curve indicating the predictive accuracy of the autophagy-related signature for CSS by using GSE13507. (G) Kaplan-Meier CSS curves for the high- and low-risk groups by using GSE19915. (H) ROC curve indicating the predictive accuracy of the autophagy-related signature for CSS by using GSE19915. (I) Kaplan-Meier CSS curves for the high- and low-risk groups by using GSE31684. (J) ROC curve indicating the predictive accuracy of the autophagy-related signature for CSS by using GSE31684. (K) Kaplan-Meier CSS curves for the high- and low-risk groups by using GSE32894. (L) ROC curve indicating the predictive accuracy of the autophagy-related signature for CSS by using GSE32894. (M) Kaplan-Meier PFS curves for the high- and low-risk groups by using GSE19915. (N) ROC curve indicating the predictive accuracy of the autophagy-related signature for PFS by using GSE19915. (O) Kaplan-Meier PFS curves for the high- and low-risk groups by using GSE32894. (P) ROC curve indicating the predictive accuracy of the autophagy-related signature for PFS by using GSE32894. (Q) Kaplan-Meier PFS curves for the high- and low-risk groups by using E-MTAB-4321. (R) ROC curve indicating the predictive accuracy of the autophagy-related signature for PFS by using E-MTAB-4321. (S) Kaplan-Meier DFS curves for the high- and low-risk groups by using TCGA-BLCA data. (T) ROC curve indicating the predictive accuracy of the autop [file Image_7.tif]

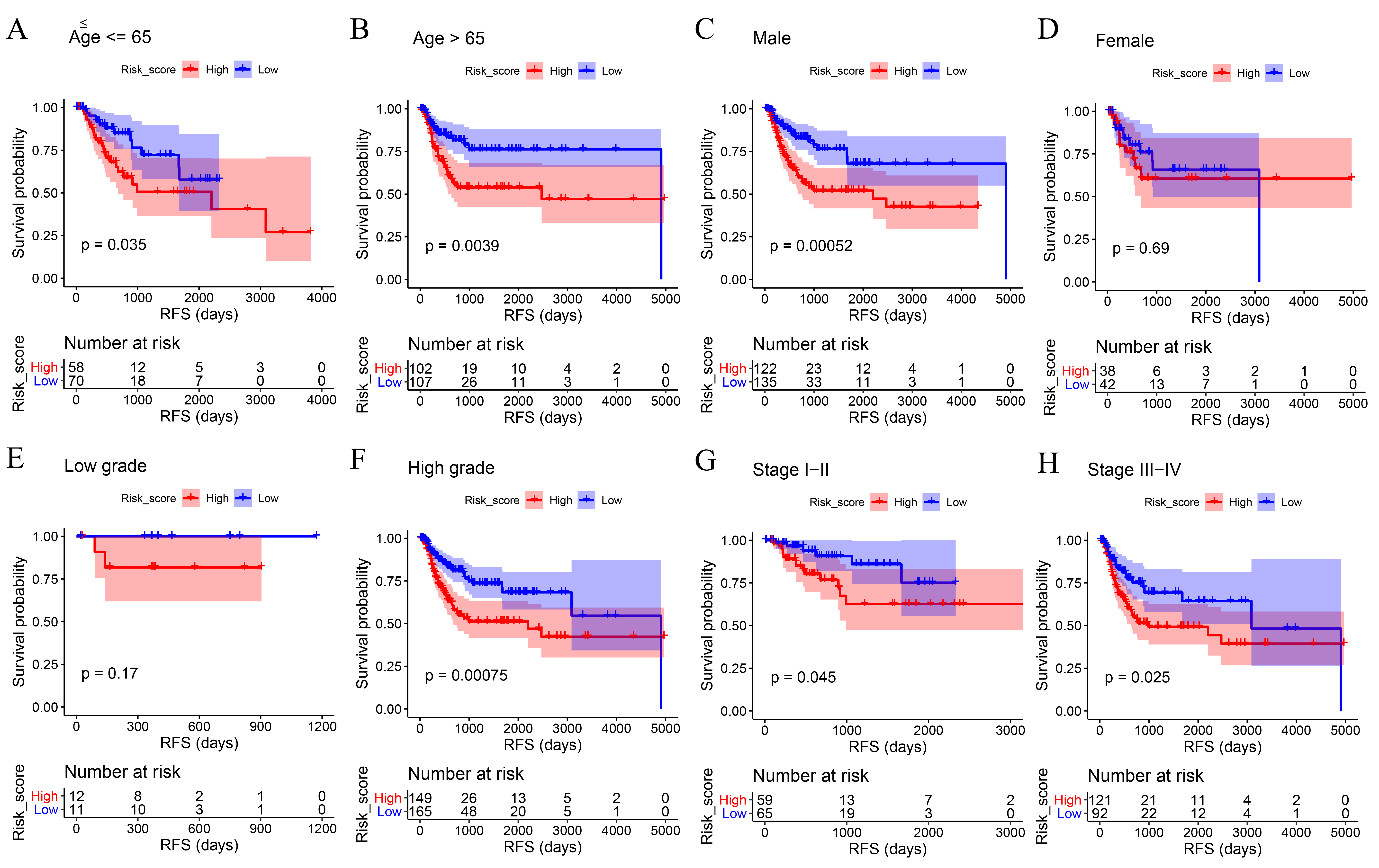

Supplement: Supplementary Figure 8 — Kaplan-Meier disease-free survival curves for the high- and low-risk groups stratified by clinicopathological variables. (A, B) Age. (C, D) Gender. (E, F) Grade. (G, H) Stage. [file Image_8.tif]

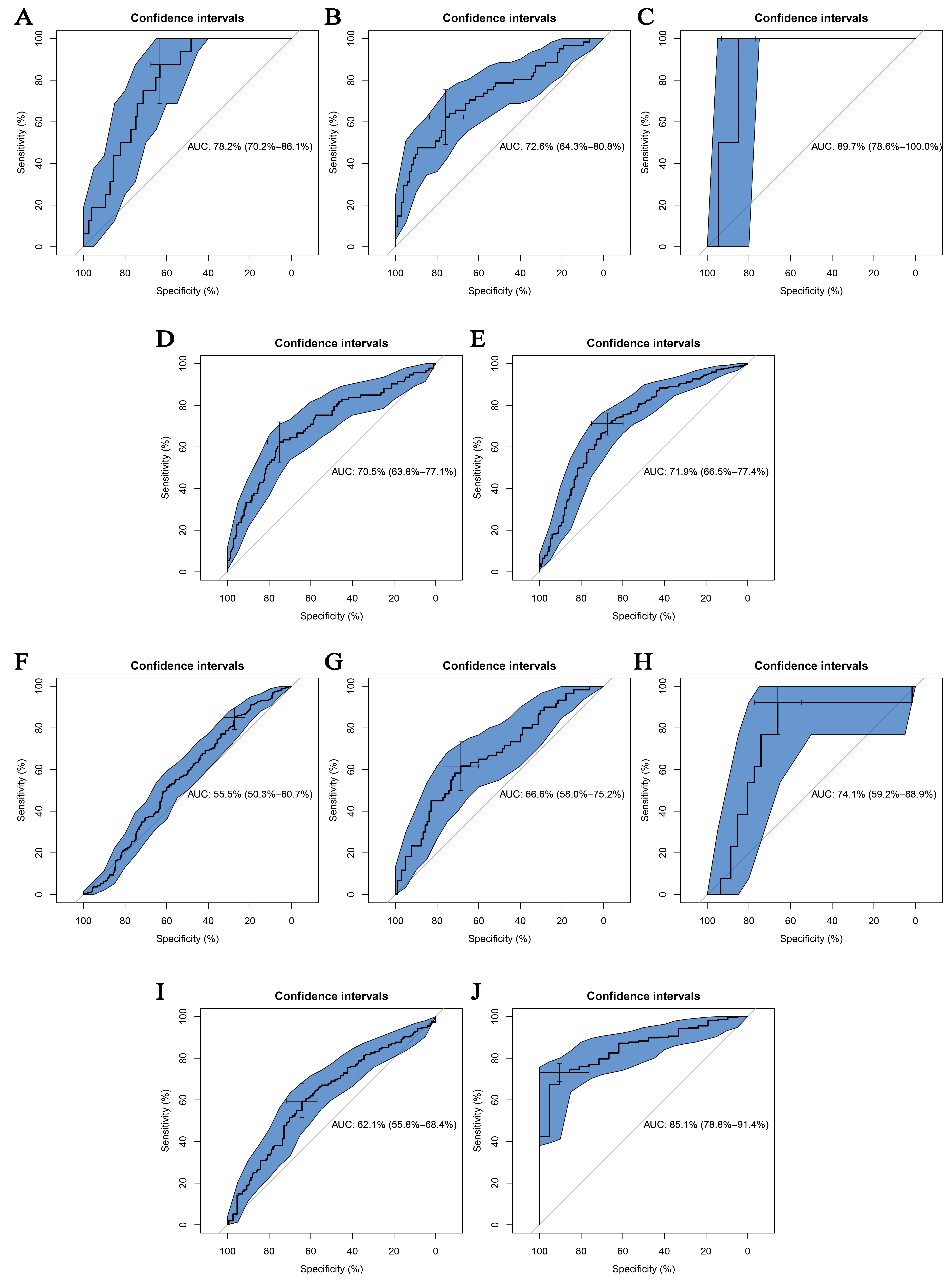

Supplement: Supplementary Figure 9 — The prognostication value of the ARG-based signature to disease stage and tumor grade by ROC curves. ROC curves to distinguish BCs of Ta-T1 stage from BCs of T2-T4 stage by using E-MTAB-4321 (A), GSE13507 (B), GSE19915 (C), GSE32894 (D). ROC curves to distinguish BCs of Ta-T1 stage from BCs of T2-T4 stage by using TCGA-BLCA data (E). ROC curves to distinguish BCs of high grade from BCs of low grade by using E-MTAB-4321 (F), GSE13507 (G), GSE19915 (H), GSE32894 (I), and TCGA-BLCA data (J). [file Image_9.tif]

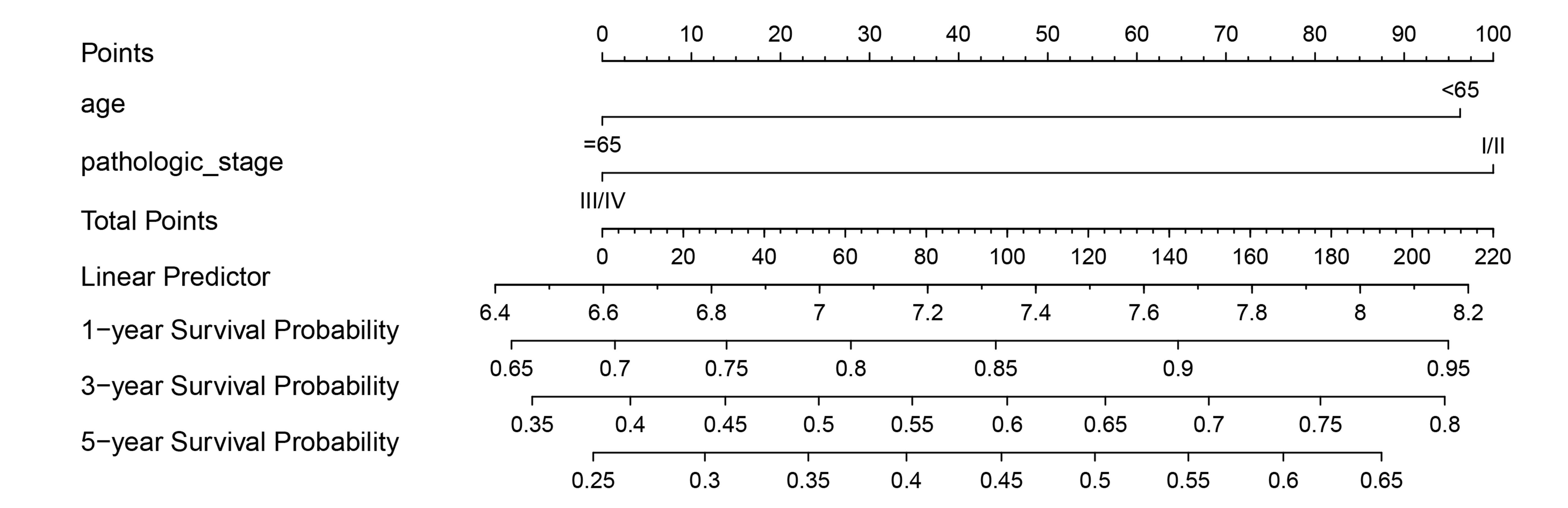

Supplement: Supplementary Figure 10 — The nomogram without the ARG signature for predicting the proportion of patients with 1-, 3- or 5-year OS. [file Image_10.tif]
